# Unusual delayed and prolonged arm weakness and atrophy following botulinum toxin injection for musician’s cramp: case report

**DOI:** 10.1007/s00702-024-02864-1

**Published:** 2025-02-17

**Authors:** Barbara I. Karp, Katharine Alter, Tanya Lehky, Mark Hallett

**Affiliations:** 1 Office of the Clinical Director, National Institute of Neurological Disorders and Stroke, National Institutes of Health, 9000 Rockville Pike, Bethesda, MD 20892 USA; 2 Department of Rehabilitation Medicine, Warren Grant Magnusen Clinical Center, National Institutes of Health, Bethesda, MD USA; 3 Electromyography Section, National Institute of Neurological Disorders and Stroke, National Institutes of Health, Bethesda, MD USA; 4 Human Motor Control Section, National Institute of Neurological Disorders and Stroke, National Institutes of Health, Bethesda, MD USA

**Keywords:** Botulinum toxin, Dystonia, Focal hand dystonia, Weakness, Atrophy, Adverse effects, Case report

## Abstract

Botulinum toxin is considered first-line treatment for focal hand dystonia in musicians. Mild, temporary weakness is a common accompaniment of effective injection. We present a unique case of delayed-onset, severe, prolonged weakness and atrophy in a patient with musician’s dystonia, successfully treated with botulinum toxin for over 10 years, following injection of his usual muscles at his well-established dose. This pianist received botulinum toxin treatment for more than 10 years, with a stable response. Six weeks after an injection, he developed progressive severe weakness and atrophy of the affected forearm involving both injected and uninjected muscles. Weakness and atrophy took over one year without further injections to resolve. The clinical course and laboratory testing were not suggestive of brachial neuritis, plexopathy, or neuralgic amyotrophy. The literature contains rare case reports of severe weakness and atrophy after botulinum toxin injection, sometimes with delayed onset and sometimes affecting distant muscles. Frequently presenting with pain, such cases often have evidence of plexopathy or neuralgic amyotrophy which were absent in our patient. Clinicians should be aware of this rare potential severe adverse event associated with botulinum toxin.

## Introduction

Over 30 years’ experience has established an excellent safety profile for botulinum toxin (BoNT) as the first line treatment of focal hand dystonia (FHD). Therapeutic response to BoNT for FHD is almost always accompanied by weakness in injected and nearby muscles directly attributable to BoNT’s disruption of acetylcholine release at the neuromuscular junction, blocking neurotransmission. The extent of weakness is proportionate to BoNT dose but does not correlate with patient benefit (Kassavetis, Lungu et al. 2020).

Although not always clinically evident, even a single injection of BoNT at a therapeutic dose can cause atrophy detectable by ultrasound and MRI (To et al. 2001, Schroeder, Ertl-Wagner et al. 2009). Clinically visible atrophy is common after repeated injections into the same muscle and is slowly reversible if injections are stopped. There have been rare reports of delayed weakness and severe atrophy with therapeutic BoNT injection. We report here a patient with musician’s cramp treated with BoNT for 10 years who developed unusual delayed onset of severe, focal arm weakness and atrophy after the most recent injection.

## Case report

A right-handed pianist first noted his 4th and 5th fingers “hanging down” when playing piano 15 years earlier. An orthopedist diagnosed trigger finger and possible carpal tunnel syndrome. EMG was normal. Cortisone injections, anti-inflammatory medications, transcutaneous electrical nerve stimulation, and acupuncture provided no relief. Two years later, he diagnosed himself with FHD.

When first seen in our clinic, he reported abnormal finger posturing when playing piano and typing. Physical examination was normal except for hyperflexion at the proximal interphalangeal joints of the right 4th and 5th fingers induced by playing piano, especially fast passages. BoNT injections were begun at a dose of 10 Units (U) onabotulinumtoxinA divided between the right flexor digitorum superficialis (FDS) fascicles to those 2 fingers. Injections continued over the next 10 years, generally at 3–6 month intervals, with the dose gradually increasing. Injection of the right flexor digitorum profundus (FDP) fascicles to fingers 4 and 5 were added after about 6 years of treatment. He had relatively stable benefit and mild-to moderate tolerable, temporary weakness with each injection cycle. His weakness was typically first noticed about 1 week after injection and lasted a mean of 11 ± 3 days.

In January 2017, he received his usual dose of 55 U onabotulinumtoxinA divided into the right FDS iv-15 Units, FDS v-15 Units, FDP iv-15 Units, and FDP v-10 Units under ultrasound guidance. When examined one month later, he had his usual slight weakness (4/5 on the MRC scale) limited to the injected finger flexors of the right hand.

Eight weeks after injection, he called to report that he had developed additional right forearm weakness over the prior 2–3 weeks that was continuing to worsen. He also noticed marked atrophy of his right medial forearm for the first time. He had no pain or sensory symptoms and no systemic complaints.

**Fig. 1:**
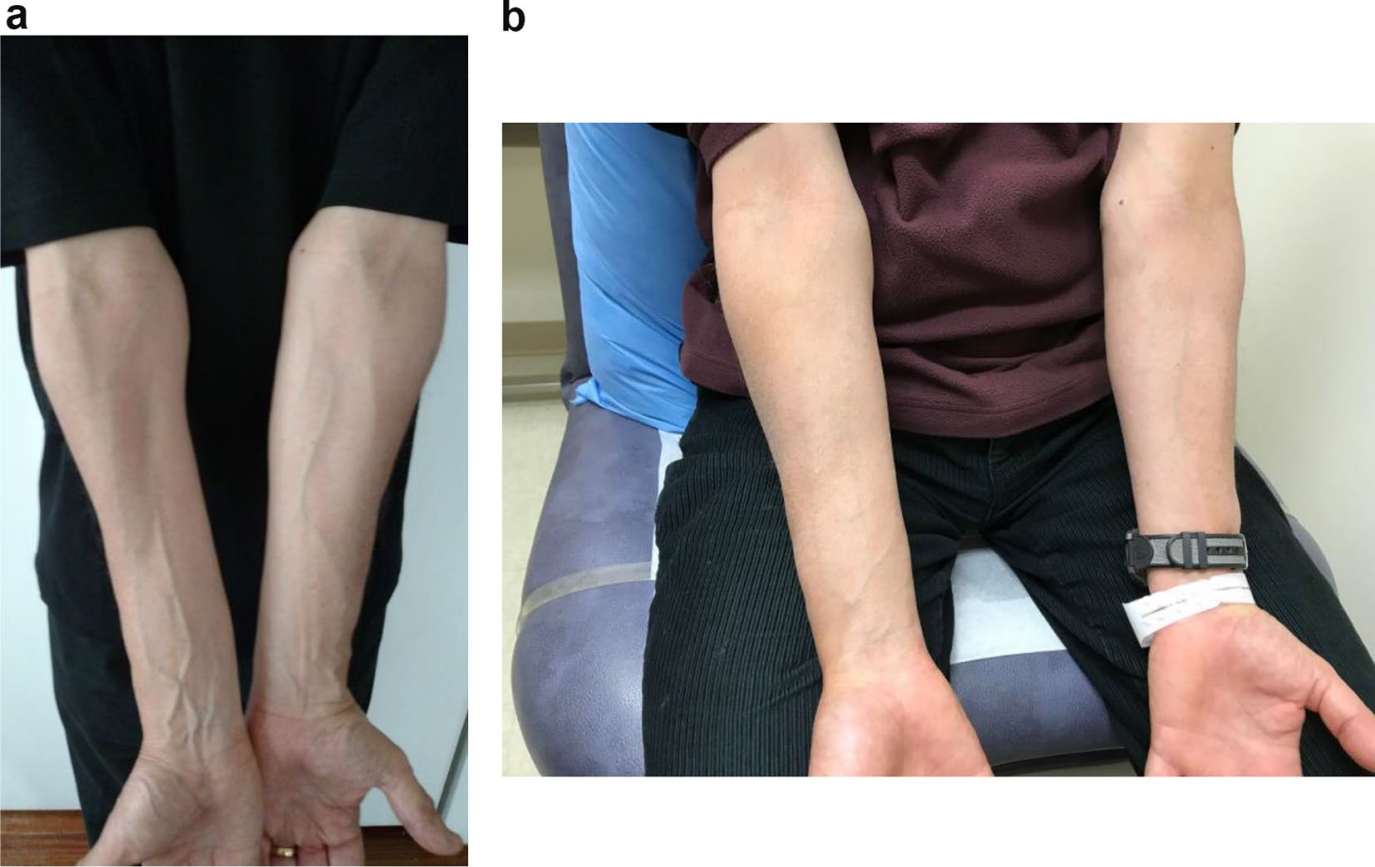
(**a**) Severe ulnar atrophy 3 months after injection of the affected right arm compared to uninjected left arm. (**b**) Partial recovery of right ulnar atrophy is apparent 7 months after injection

He was unable to return for evaluation until 3 months after injection. Examination then revealed marked atrophy of the right ulnar forearm [Fig. 1a], with lesser atrophy of the hypothenar eminence.

**Fig. 2:**
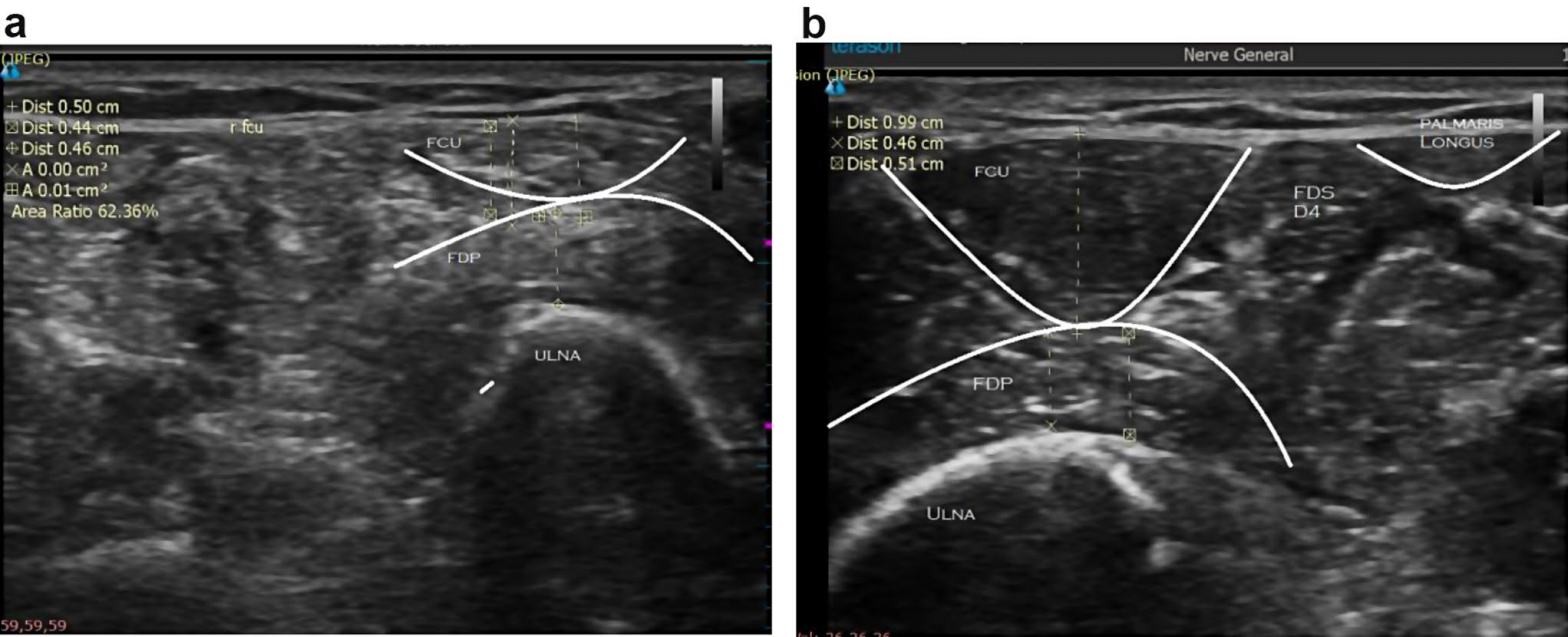
(**a**) Ultrasound shows atrophy of the flexor carpi ulnaris muscle (width 0.50 cm) in the affected side 3 months after injection. (**b**) Ultrasound of normal flexor carpi ulnaris muscle (width 0.99 cm) in the unaffected side 3 months after injection

Atrophy was apparent on ultrasound [Fig. 2a, b].

The FDS and FDP were weaker than on the prior examination 1 month after injection. Weakness was also present in uninjected muscles including pronator teres (PT), wrist flexors and abductor digiti quinti (ADQ) in the injected right arm. There was no proximal right arm weakness and strength was normal in the left arm and both legs. Biceps, brachioradialis and triceps reflexes were present and symmetric. Sensory examination and blood tests including ESR, CRP, and CK, were normal. The initial electrodiagnostic findings, at 4 months after the botulinum injection, showed normal right median and ulnar nerve motor and sensory nerve conduction studies with normal radial, medial cutaneous nerve of the forearm and lateral cutaneous nerve of the forearm sensory nerve studies (Table 1). Needle EMG studies showed evidence of active denervation with the presence of fibrillations and positive sharp waves in 3 muscles: 2 ulnar innervated muscles (flexor carpi ulnaris and abductor digiti minimi) and 1 median innervated muscle (pronator teres). The ulnar-innervated first dorsal interossei, median-innervated abductor pollicis brevis, and radial innervated brachioradialis, extensor indicis propius and triceps muscles were normal as were the right low cervical paraspinal muscles. The normal nerve conduction studies and the distribution of the needle EMG findings did not suggest an isolated neuropathy, brachial plexus lesion or cervical radiculopathy.

**Table 1:** Diagnoses and symptomatology of reported cases of prolonged/delayed severe weakness and atrophy after botulinum toxin injection

Reference	Sex/Age Bracket	Diagnosis	Duration of Prior BoNT Treatment	Onset pain/weakness (time from injection)	Duration weakness/time to improvement	Pain
Present case	M, 60s	MC	10 years	Pain: none Weakness: 6 weeks	at least 1 year	no
Alcalay 2010	F, 60s	CD	1st injection	Pain: 8 days Weakness: 1 month	4 months	yes
Burguera 2000	M, 40s	CD, MFD	16 months	Pain/discomfort: 6 weeks Weakness: 6 weeks	3 months	yes
Cani 2019 case 1	F, 70s	CD	14 years	Pain: 8 weeks Weakness: 8 weeks	Improving at 10 weeks	yes
Cani 2019 case 2	M, 70s	CD/ tremor	4 years	Pain: 8 weeks Weakness: onset not stated, present at week 8	Began to recover 6–8 months	yes
Cani 2019 case 3	M, 70s	CrCD	2 years	Pain: not stated Weakness: 6–8 week	Partial recovery by 8–9 months	yes
Cani 2019 case 4	F, 60s	WC, CrFD	5years	Pain: not stated Weakness: 2–3 weeks	Complete recovery by 4–5 months	yes
Eickhoff 2016	F, 40s	SLE/ Raynaud’s	1st injection	Pain: none Weakness: 2 days	Functional improvement but not at premorbid function 2 years later	no
Glanzman 1990	F, 50s	CD	1st injection	Pain: 2 days Weakness: 2 days	Partial recovery by 5 months	yes
Glass 2009	F, teens	PH	2 years	Pain: none. Weakness: not stated	Partial recovery at 3 months	no
Sampaio 1993	F, 30s	CD	1st injection	Pain: 6 days after first injection Weakness: 2–3 days after booster injection	6 weeks	yes
Sheean 1995 case 1	F, 30s	WC	1st injection	Pain: 7 days after first injection. Weakness: 2–3 days after booster injection	Partial recovery by 3 months	yes
Sheean 1995 case 2	F, 50s	WC, SD	18 months WC; 22 months SD	Pain: 1 week after session 1 Weakness: 1 week after session 2	Start of improvement at 6 weeks; partial recovery by 12 weeks	yes
Tarsy 1997	M, 50s	CD	1st injection	Pain: none. Weakness: 10 days	Complete recovery by 7 months	no
Zhao 2012	F, 20s	HA	1st injection	Pain: none. Corrugator atrophy: 10 weeks	Partial recovery 6 months; complete recovery 1 year	no

By 7 months after injection, atrophy and weakness appeared to be resolving. He was able to play piano for about 1 h/day. The repeat needle EMG showed continued presence of fibrillations and positive sharp waves with an evolving reinnervation pattern of polyphasic and larger motor units in the flexor carpi ulnaris, pronator teres, flexor digitorum profundus and flexor digitorum superficialis. The abductor digiti minimi showed slight increase in motor unit size consistent with a reinnervation pattern.

Muscle strength was almost normal 11 months after injection with only slight weakness of right hand finger flexion. Muscle bulk showed marked improvement although residual atrophy was still apparent [Fig. 1b]. The needle EMG studies showed similar findings to the prior study with continued the presence of fibrillations and positive sharp waves and evolving reinnervation pattern of polyphasia and larger motor units. He reported that his focal hand dystonia had fully returned as well. When last seen 14 months after injection, he had only minimal residual weakness of right 4th finger flexion. The EMG showed almost complete resolution of fibrillations and positive sharp waves with continued evidence of evolving reinnervation in the FCU, FDP, FDS and PT.

## Discussion

Our patient had an unusual course of severe atrophy along with delayed and prolonged weakness following an injection of BoNT for FHD, despite having received injections over the prior 10 years without such a complication. Weakness was present not only in injected (FDS, FDP) and uninjected adjacent (PT, FCU, FCR) muscles, but also in the distal, uninjected muscle ADQ. Unlike his typical weakness which began within a week of injection and lasted a mean of 11 days after injection, the severe weakness began later, about 6 weeks after injection, and atrophy lasted over a year in the absence of further BoNT injection. The electrodiagnostic studies show that the distribution of denervated muscles were all in close proximity to the botulinum injection but not in the same nerve or cord distribution, making an isolated neuralgic amyotrophy unlikely. Similarly, sensory nerve responses, which are frequently abnormal in brachial plexus lesions were all spared.

Animal and human studies have shown that even a single BoNT injection can cause muscle atrophy persisting for months, although such atrophy is often not clinically apparent (Ansved, Odergren et al. 1997, Schroeder, Ertl-Wagner et al. 2009). Type IIB fiber trophy has also been observed on biopsy of muscles distant from injection site (e.g., vastus lateralis) following repeated injections for cervical dystonia (Ansved, Odergren et al. 1997). Similarly, increased jitter on single-fiber EMG was detected in the extensor digitorum muscles of patients who received BoNT for blepharospasm or hemifacial spasm (Girlanda, Vita et al. 1992).

Severe, prolonged weakness and atrophy, including that in muscles distant from the sites of injection and sometimes with delayed onset, have not been reported in large-scale clinical trials of BoNT. However, the literature contains rare case reports with similar elements [Tables 2 and 3]. The affected patients in these reports included both men and women being injected for a variety of indications and with type A toxins, onabotulinumtoxinA or abobotulinumtoxinA. Some patients developed such complications with the first injection; in some patients, the pronounced weakness followed a “booster” injection. Others (like our patient) had received repeated injection sessions of BoNT over many years.

**Table 2:** Botulinum toxin usage, injection sites, weakness distribution and diagnoses in reported cases of prolonged/delayed severe weakness and atrophy after botulinum toxin injection

Reference	drug	dose (units)	injection site/s	location of weakness	Diagnosis by author	Later injection
Present case	ona	55	R FDS, R FDP	FDS, FDP, PT, FCR, FCU, ADQ	direct effect of toxin	not stated
Alcalay 2010	ona	150	L SCM, R SC	internal rotation and abduction arm	neuralgic amyotrophy involving upper trunk of plexus	no
Burguera 2000	ona	200	SC, Trap	generalized ascending weakness	acute demyelinating polyradiculoneuritis	no
Cani 2019 case 1	type A NOS	600	R/ L SC, R SCM, R/L LS	arm abduction	brachial neuritis	not stated
Cani 2019 case 2	type A NOS	900	R/L SC	shoulder girdle	brachial neuritis	yes
Cani 2019 case 3	type A NOS	570	LSC, L LS, R/L SCM, R/L LAO	shoulder abduction	brachial neuritis with partial lesion of plexus upper trunk vs. C5 motor radiculopathy	not stated
Cani 2019 case 4	type A NOS	100	R/L OO	shoulder abduction, int/ext rotation, elbow flex/ext, palmar flexion, thumb abduction	brachial neuritis	yes
Eickhoff 2016	ona	100	base of fingers	thenar atrophy, weak grip	direct effect of toxin	not stated
Glanzman 1990	not stated	120	L SCM, L trap	deltoid, biceps, infraspinatus, supraspinatus	brachial plexopathy	not stated
Glass 2009	abo	500/ palm	palms	thenar and hypothenar atrophy	direct effect of toxin	not stated
Sampaio 1993	abo	800 initial; 400U booster 2 weeks later	SCM, trap, SC	supraspinatus, deltoid, biceps	partial bilateral brachial plexopathy	not stated
Sheean 1995 case 1	abo	120 over 2 sessions	FPL, FDP	supinator, wrist extension, brachioradialis, triceps, intrinsic hand muscles	neuralgic amyotrophy	not stated
Sheean 1995 case 2	abo	300 over 2 sessions	FCR, ECR, ECU, FCU	wrist drop, weakness arm and hand	neuralgic amyotrophy	no
Tarsy 1997	type A NOS	160	SC, LS, SCM	arm abduction	brachial plexopathy of upper trunk	yes
Zhao 2012	ona	10 each corrugator, 185U total for headache	corrugators	corrugators	direct toxin effect	yes

Diagnosis-MC: musician’s cramp, WC: writer’s cramp, CD: cervical dystonia, MFD: multifocal dystonia, Cr/CD: craniocervical dystonia, Cr/FD: craniofacial dystonia, SLE: systemic lupus erythematosus, PH: palmar hyperhidrosis, SD: spasmodic dysphonia, HA: headache

Drug- Ona: onabotulinumtoxinA, Abo: abobotulinumtoxinA, NOS: not otherwise specified

Injection sites/Location of weakness: R:right, L:left

ADQ: abductor digiti minimi, ECR: extensor carpi radialis, ECU: extensor carpi ulnaris

FCR: flexor carpi radialis, FCU: flexor carpi ulnaris, FDP: flexor digitorum profundus, FDS: flexor digitorum superficialis, FPL: flexor pollicis longus, LAO: levator anguli oris, LS: levator scapuli, OO: orbicularis oculi, PT: pronator teres, SC: splenius capitus, SCM: sternocleidomastoid, Trap: trapezius

**Table 3:** Electrodiagnostic nerve conductiontesting

	Time after BoNT injection	Motor				Sensory	
		DL	Amp	CV	F-wave	Amp	CV
R Median	4 mo	2.86	6.0	51.8	32.1	25.9	56
	5 mo	3.75	7.3	53.1	30.8	21.6	57.2
	7 mo	3.28	5.6	52.5	29.9	20.6	54.9
	10 mo	3.7	8.1	55	30.8	20.7	56.4
	13 mo	3.54	7.6	50.5	31.9	21.1	54.9
R Ulnar	4 mo	2.40	7.6	57.6	30.5	11.3	52.4
	5 mo	2.34	8.6	60.0	29.4	15.4	60.6
	7 mo	2.71	8.7	58.7	31.6	10.5	51.5
	10 mo	2.4	8.6	60.3	30.5	19.1	50.1
	13 mo	2.24	8.3	56.4	31.3	19.9	56.2
R Radial	4 mo					10.1	61.9
R LCN	4 mo					11.4	68.9
R MCN	4 mo					8.6	80.0

Amp: amplitude (motor- millivolts; sensory-microvolts), DL: distal latency (msec), CV: conduction velocity (meters/sec)

F-wave-milliseconds, LCN: lateral cutaneous nerve of forearm, MCN: medical cutaneous nerve of forearm

In a number of the reported cases, patients had prominent pain early in the course followed by weakness and atrophy affecting muscles distant from those injected, at times including contralateral or bilateral musculature (Glanzman, Gelb et al. 1990, Sampaio, Castro-Caldas et al. 1993, Sheean, Murray et al. 1995, Tarsy 1997; Burguera, Villaroya et al. 2000, Alcalay, Simoes et al. 2010, Cani, Latorre et al. 2019). Such cases were commonly associated with clinical examination and NCV/EMG evidence of plexopathy, polyradiculoneuropathy or neuralgic amyotrophy, conditions often attributed to inflammatory or immune-mediated processes as they are commonly associated with antecedent immunization or infection. Although, given the rarity of occurrence following BoNT injections and the frequency of these conditions in general, the relationship may be coincidental (Vieregge and Kompf 1993). Cani et al. identified 4 cases among 460 patients receiving injections over a 10 year period at their center (Cani, Latorre et al. 2019), and Sheean et al. encountered 2 cases in a clinic that had treated only about 100 patients with upper limb dystonia (Sheean, Murray et al. 1995), while the incidence of brachial neuritis in the general population has been reported at 1 per 1000 person-years. (van Alfen, van Eijk et al. 2015)

In the cases of distant or delayed weakness classified as plexopathy, polyradiculoneuropathy or neuralgic amyotrophy, pain was prominent and often preceded the weakness. Affected muscles were often those in the proximal upper extremity and/or shoulder girdle. Several of patients with presumed immune-mediated plexopathy or neuralgic amyotrophy went on to resume BoNT injection without recurrence (Tarsy 1997; Zhao and Stillman 2012; Cani, Latorre et al. 2019).

In contrast, our patient had prolonged painless weakness and atrophy limited to the injected limb, including involvement of some muscles distal to the injection. Brachial neuritis is painless in about 4% of patients (van Alfen and van Engelen 2006). While the absence of pain or sensory symptoms and distal distribution of weakness largely in injected and adjacent muscles in our patient make that diagnosis less likely, it cannot be definitively ruled out. Glass et al. reported a case similar to ours: a patient who received palmar and plantar injections of abobotulinumtoxinA for hyperhidrosis for 2 years before developing severe, prolonged hand intrinsic muscle weakness and atrophy. Without signs of nerve compression, radiculopathy or plexopathy, the symptoms were believed to be a direct effect of the toxin (Glass, Hussain et al. 2009). Zhao et al. described a patient with late onset (10 weeks after injection) and prolonged (recovery over 1 year) corrugator supercilii atrophy following injection for headache (Zhao and Stillman 2012). In these and our case, weakness and atrophy appear to be a direct effect of toxin on the neuromuscular junction and muscle. Without further toxin injection, strength and muscle bulk slowly recover, so at least it appears that the mechanism is monophasic. The patient described by Zhao et al. continued BoNT for headaches without injection of the corrugators and no further complications (Zhao and Stillman 2012). It is not known if toxin can be safely restarted, with injection in the previously injected muscles, in such patients. Neither we nor our patient were eager to pursue further injections.

## Data Availability

This paper is a case report of a single patient. There is no data associated with it. Patient testing results are in the tables within the paper itself.
